# SLC27A2 is a potential immune biomarker for hematological tumors and significantly regulates the cell cycle progression of diffuse large B-cell lymphoma

**DOI:** 10.1186/s12920-024-01853-3

**Published:** 2024-04-25

**Authors:** Yi Wang, Xue Chen, Yun Li, Zhixue Zhang, Leiming Xia, Jiang Jiang, Yuqin Chai, Ziming Wang, Yu Wan, Tongyu Li, Fengbo Jin, Hongxia Li

**Affiliations:** 1 Department of Oncology, The Third Affiliated Hospital of Anhui Medical, Anhui, China; 2 Graduate School Internal Medicine, Bengbu Medical College, Anhui, China; 3 Kindstar Global Precision Medicine Institute, Wuhan, China; 4 Department of Scientific Research Project, Wuhan Kindstar Medical Laboratory Co., Ltd, Wuhan, Hubei China; 5 Department of Hematology, The Ji’an Central Hospital, Jiangxi, China; 6 Department of Hematology, The First Affiliated Hospital of Anhui Medical, Anhui, China; 7 Department of Ophthalmology, The First Affiliated Hospital of Anhui Medical, Hefei, Anhui China; 8 Ningbo Clinical Research Center for Hematologic Malignancies, the First Affiliated Hospital of Ningbo University, Ningbo, Zhejiang China

**Keywords:** DLBCL, SLC27A2, Fatty acid metabolism, Cell cycle, Apoptosis

## Abstract

**Background:**

Research on the fatty acid metabolism related gene SLC27A2 is currently mainly focused on solid tumors, and its mechanism of action in hematological tumors has not been reported.

**Method:**

This study aims to explore the pathological and immune mechanisms of the fatty acid metabolism related gene SLC27A2 in hematological tumors and verify its functional role in hematological tumors through cell experiments to improve treatment decisions and clinical outcomes of hematological tumors.

**Result:**

This study identified the fatty acid metabolism related gene SLC27A2 as a common differentially expressed gene between DLBCL and AML. Immune microenvironment analysis showed that SLC27A2 was significantly positively correlated with T cell CD4 + , T cell CD8 + , endothelial cells, macrophages, and NK cells in DLBCL. In AML, there is a significant negative correlation between SLC27A2 and B cells, T cell CD8 + , and macrophages. SLC27A2 participates in the immune process of hematological tumors through T cell CD8 + and macrophages. The GESA results indicate that high expression of SLC27A2 is mainly involved in the fatty acid pathway, immune pathway, and cell cycle pathway of DLBCL. The low expression of SLC27A2 is mainly involved in the immune pathway of AML. Therefore, SLC27A2 is mainly involved in the pathological mechanisms of hematological tumors through immune pathways, and cell experiments have also confirmed that SLC27A2 is involved in the regulation of DLBCL cells.

**Conclusion:**

In summary, our research results comprehensively report for the first time the mechanism of action of SLC27A2 in the immune microenvironment of DLBCL and AML, and for the first time verify the cycle and apoptotic effects of the fatty acid related gene SLC27A2 in DLBCL cells through cell experiments. Research can help improve the treatment of AML and DLBCL patients.

**Supplementary Information:**

The online version contains supplementary material available at 10.1186/s12920-024-01853-3.

## Introduction

Diffuse large B-cell lymphoma(DLBCL) is one of the most common types of adult lymphoma and a subset of malignant tumors, with high heterogeneity in clinical manifestations and prognosis [1, 2]. DLBCL is the most common malignant tumor of the lymphatic system in adults, accounting for almost 35–40% of lymphoma in Western countries and usually higher than 40% in Asian countries [3]. Most patients do not have obvious clinical symptoms, but about one-third of them experience symptoms such as fever, night sweats, and weight loss, which are mostly related to the site of tumor infiltration. In recent years, chemotherapy and immunotherapy have further improved the prognosis of DLBCL [4, 5]. However, only a small number of DLBCL patients responded well to this treatment [6]. Therefore, there is an urgent need to find new biomarkers to improve patient treatment.

Tumor infiltrating immune cells are associated with DLBCL. Tumor cells are usually colonized in normal tissues and can form tumor microenvironment (TME) together with immune cells and their secreting factors, stromal cells, vascular endothelial cells, and extracellular matrix components [7]. In the early stages of tumor progression, the main cellular components that maintain the immunosuppressive microenvironment exert anti-tumor effects. Targeting TME can stimulate or restore the inherent tumor suppressive ability of the immune system and reshape a positive immune microenvironment [8, 9]. However, the heterogeneity of TME allows for widespread differences in tumor progression between individuals. Therefore, a deeper understanding of the tumor immune microenvironment will help us improve the treatment of DLBCL patients [10]. A study has found that higher serum free fatty acid levels before treatment are associated with lower survival rates in untreated DLBCL patients [11]. Another study found that exposure to omega-3 fatty acids docosahexaenoic acid at clinically achievable doses can significantly alter the whole genome level of histone post-translational modifications in DLBCL cells, which is a cell line dependent and dose dependent approach [12]. Therefore, fatty acids may play an important role in the prognosis and pathological mechanisms of DLBCL.. Fatty acids are an important component of lipid metabolism, playing a crucial role in the pathogenesis of tumors and regulating tumor immunity [13, 14]. However, the mechanism of action of these fatty acid metabolism related genes in DLBCL has not been elucidated, and the mechanism of action of the key fatty acid metabolism gene SLC27A2 in DLBCL has not been reported.

This study systematically analyzed the expression patterns and immune microenvironment mechanisms of fatty acid metabolism related genes in DLBCL. Then, the functional role of the fatty acid metabolism related hub gene SLC27A2 in DLBCL cells was verified through cell experiments. And further evaluated the potential mechanism of action of SLC27A2 at the protein level.

## Materials and methods

### Data collection and preprocessing

Two independent gene expression profiles of diffuse large B-cell lymphoma, GSE56315 and GSE25638, from the Gene Expression Comprehensive Database (GEO) (https://www.ncbi.nlm.nih.gov/geo/). Among them, the GSE56315 dataset was obtained by the GPL570 platform, including 55 DLBCL samples and 33 matched normal samples. The GSE25638 dataset was obtained from the GPL570 platform, including 26 DLBCL samples and 13 matched normal samples. The GSE10846 dataset was obtained by the GPL570 platform, including 181 clinical samples from CHOP(Cyclophosphamide, Hydroxydaunomycin, Oncovin and Prednisone) treated patients and 233 clinical samples from patients treated with rituximab CHOP. The GSE181063 dataset was obtained from the GPL14951 platform, including 1311 formalin fixed paraffin embedded (FFPE) samples of confirmed DLBCL. The acute myeloid leukemia dataset comes from GSE30029 and TCGA-LAML, the TCGA dataset can be downloaded from the Cancer Genome Atlas (TCGA) database (https://www.cancer.gov/ccg/research/genome-sequencing/tcga).The GSE30029 dataset was obtained from the GPL6947 platform, including 90 AML samples and 31 matched normal samples. We used the R 4.2.1 tool to evaluate gene expression levels and prognosis by analyzing raw data from microarrays [15]. The TCGA-DLBC dataset includes 33 DLBCL samples and the Genotype-Tissue Expression (GTEx) dataset includes 337 normal samples to verify the expression of key hub genes, The TCGA-LAML dataset includes 173 AML samples and the GTEx dataset includes 70 normal samples to verify the expression of key hub genes [16].

### Expression and mechanism analysis of fatty acid metabolism related genes in DLBCL

To evaluate the expression level of fatty acid metabolism related genes in DLBCL, GEO data GSE56315 and GSE25638 were used for gene expression data analysis in DLBCL. Specifically, after data standardization, a box plot is created, with rows representing the sample and columns representing the gene expression values in the sample. If there is no batch effect in the data, it can be used as a batch of data for subsequent analysis. Fold change and corrected *p*-values (FC > 1.30 or < 0.77 and *P *< 0.05 are considered to have significant differences in genes) are used to screen for differentially expressed genes [17]. The data of fatty acid metabolism related genes were extracted from databases such as the Kyoto Encyclopedia of Genes and Genomes (KEGG), Hallmark, and Reactome pathways. Next, we used Venn plots and Gene Ontology (GO) enrichment analysis to explore the molecular mechanisms of fatty acid related differentially expressed genes in DLBCL [18].

### Functional and pathway enrichment analysis

Metascape (https://metascape.org/gp/index.html#/main/step1) It is a website for analyzing gene or protein lists, used to analyze functional clustering of gene sets. The R package ClusterProfiler package is used to analyze the GO and the gene set of the KEGG, and P < 0.05 is considered significant. Conduct Gene Set Enrichment Analysis (GSEA) to investigate the biological signaling pathways between high and low key Hub gene expression [19].

### Analysis of key fatty acid Hub genes in DLBCL

Utilizing the STRING database (https://string-db.org/) Obtain the network relationship diagram of differentially expressed fatty acid related genes and obtain the Hub gene module through the MCODE plugin in Cytoscape software [20].

### Validation of expression analysis of key Hub genes in DLBCL

To verify the expression level of key Hubs, we obtained data from TCGA-DLBC and GTEx datasets and conducted gene expression analysis. After data standardization, a block diagram will be created where rows represent the samples and a list shows the gene expression values in the samples. If there is no batch effect in the data, it can be used as a batch of data for subsequent analysis. The red color in the figure represents DLBCL samples, while the black color represents healthy control samples. It is believed that there are significant differences in genes [15].

### Construction of cell model for SLC27A2 interference

SiRNA is a chemically synthesized small molecule that serves as an important intermediate for gene silencing and sequence specific RNA degradation. It has special structural features such as a 5 'end phosphate group and a 3' end hydroxyl group, with two free bases at the 3 'end of each of its two chains. It degrades mRNA through specific complementary binding with the target mRNA. We constructed three siRNAs (SLC27A2 siRNA1, SLC27A2 siRNA2, and SLC27A2 siRNA3) based on the SLC27A2 sequence. After transfection into OCI-LY1 cells, the interference effect of these three siRNAs on SLC27A2 was verified through qPCR. A random sequence was used as the control group (SLC27A2 NC), and the cell model of SLC27A2 interference was constructed by selecting one of the three siRNAs with the best interference effect and significant interference effect [21].

### Cell cycle experiment

Firstly, discard the culture medium, wash the cells with 2 ml PBS, add 1 ml trypsin, digest in a 37 ℃ incubator for 1 min, terminate digestion with the medium, collect the cell suspension, centrifuge at 1000 rpm for 5 min, discard the supernatant, add 5 ml of pre-cooled 70% ethanol, mix well while adding, and fix overnight at 4 ℃. Then collect the cells by centrifugation, wash them twice with 5 mL of PBS, centrifuge at 1000 rpm for 5 min, discard the supernatant, and add 500uL of permeable solution (100 μ G/mL RNase A, 0.2% Triton X-100) incubated at 37 ℃ for 30 min. Subsequently, wash the cells twice with 5 mL of PBS, 50 μ Dye with g/mL propidine iodide for 10 min, pass through a 300 mesh sieve, centrifuge at 1000 rpm for 5 min, and discard the supernatant. Finally, add 5 ml PBS to wash off excess propidine iodide and use 200 μ After resuspension of PBS cells, flow cytometry detection was performed [22].

### Cell apoptosis experiment

First, collect cells (1 × 106 pieces/time), wash with cold PBS, and then use 1 ml 1 × Binding Buffer suspension cells, 300 × Centrifuge for 10 min and discard the supernatant. Using 1 ml 1 × The Binding Buffer resuspended cells to achieve a cell density of 1 × 106 pieces/ml. Then add 100 to each tube μ L cells (1 × 105) and 5 μ L Annexin V-FITC, gently mix at room temperature and dark conditions for 10 min, then add 5 μ L propidine iodide, room temperature, dark, incubate for 5 min.

Finally, add PBS to 500 μ L. Gently mix and detect within 1 h using a flow cytometry [23].

### Validation of expression analysis of key Hub genes in AML

To verify the expression level of key Hubs, we obtained data from TCGA-LAML, GTEx datasets and GSE30029 and conducted gene expression analysis. After data standardization, a block diagram will be created where rows represent the samples and a list shows the gene expression values in the samples. If there is no batch effect in the data, it can be used as a batch of data for subsequent analysis. The red color in the figure represents AML samples, while the black color represents healthy control samples. It is believed that there are significant differences in genes [15].

### Correlation analysis of clinical information between SLC27A2 and DLBCL and AML

Download clinical information of DLBCL and AML patients from TCGA and analyze the correlation between SLC27A2 and patient analysis and age through expression differences [19].

### Correlation analysis between immune microenvironment and SLC27A2 in DLBCL and AML

We used EPIC to analyze the correlation between SLC27A2 and a large number of tumor infiltrating immune cells in DLBCL and AML. Spearman correlation analysis between SLC27A2 and immune score. The horizontal axis in the figure represents the expression or model score distribution of the first gene, the vertical axis represents the immune score distribution, and the density curve on the right represents the trend of immune score distribution; The upper density curve represents the distribution trend of a gene or model score; The top numerical value represents the correlation p-value, correlation coefficient, and correlation calculation method [24].

### Protein interaction analysis of key Hub Genes in DLBCL and AML

To investigate the protein interaction mechanism of key Hub genes in DLBCL, we used GeneMANIA (https://genemania.org/) constructed a PPI network centered around key Hub genes was constructed, which includes association data on protein genetic interactions, pathways, co expression, co localization, and protein domain similarity. Then, GO functional enrichment and KEGG pathway analysis were performed on genes constructed using GeneMANIA centered around the Hub gene [19].

### Statistical analysis

As mentioned above, most statistical analyses of differential gene expression were conducted using R 4.2.1 and online databases [15]. Perform statistical analysis on data obtained from the database using GraphPad Prism software version 7.0 (GraphPad Software Inc., La Jolla, California). Student t-test or rank sum test is used for comparison between two groups. Survival analysis was conducted using the Kaplan Meier method and logarithmic rank test [25].

## Results

### Expression and mechanism analysis of fatty acid metabolism related genes in DLBCL

**Fig. 1:**
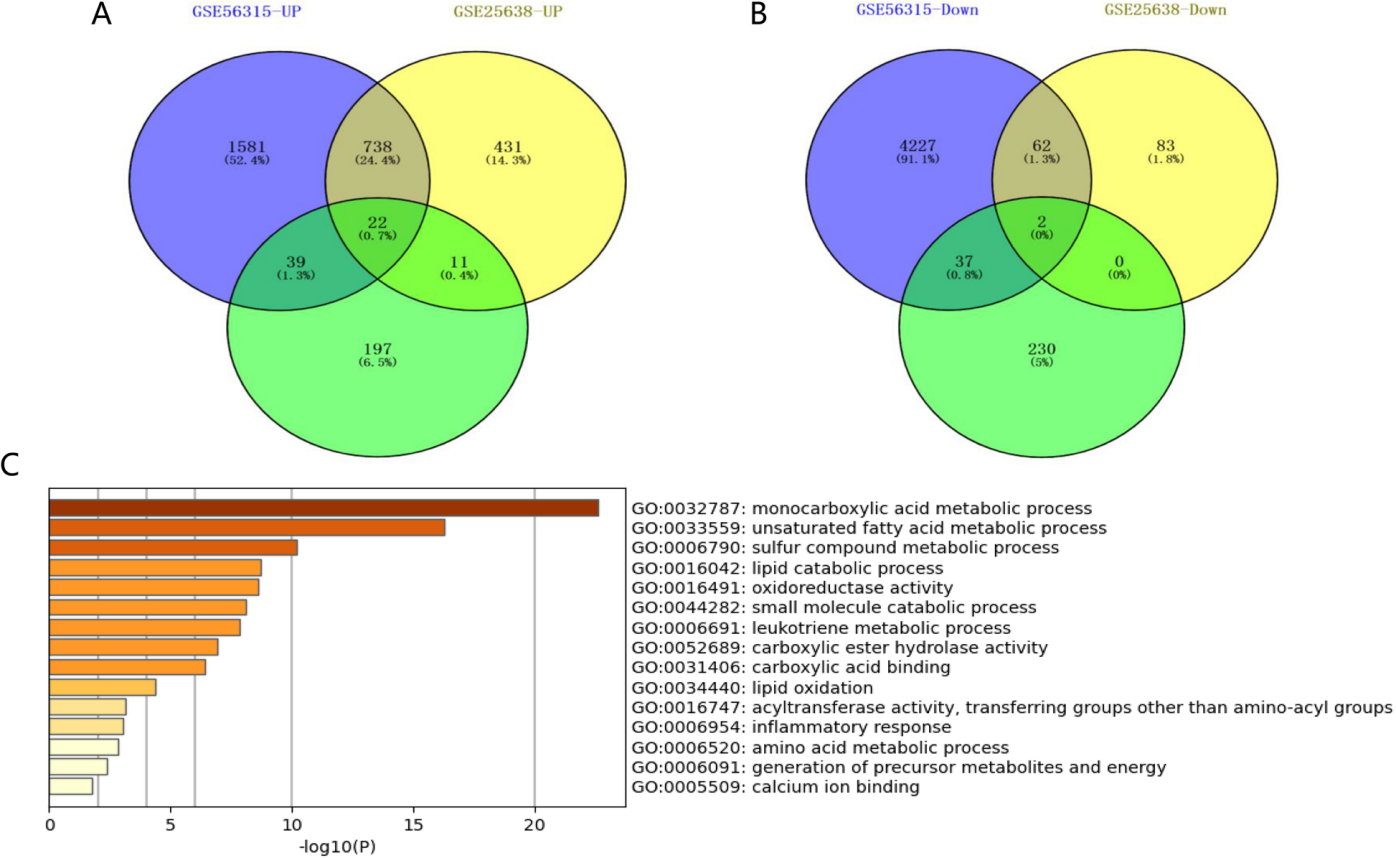
Screen and mutually validate fatty acid related genes with significant differences in expression using the GSE56315 dataset and GSE25638 dataset, and study their functions through GO enrichment analysis**.** A total of 24 fatty acid metabolism related genes were screened in the DLBCL dataset GSE56315 and GSE25638, of which 22 were upregulated (**A**) and 2 were downregulated (**B**). 24 fatty acid metabolism related genes are mainly enriched in the GO pathway, including Monocarboxylic acid metabolic process, Unsaturated fatty acid metabolic process, Sulfur compound metabolic process, Lipid catabolic process, and Oxidoreductase activity (**C**)

**Fig. 2:**
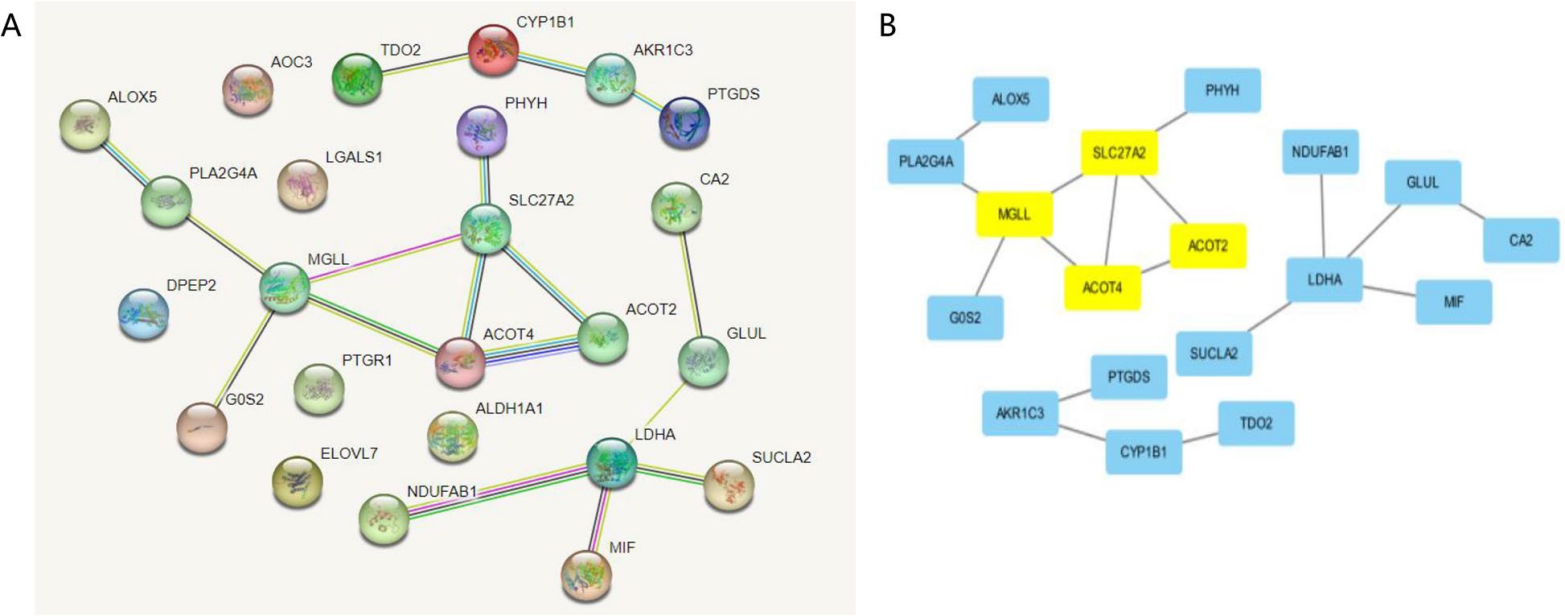
Build a PPI network in the STRING database, use the MCODE plugin in Cytoscape to filter key subnets and visualize them. Building a PPI network (**A**) in the STRING database, SLC27A2, MGLL, ACOT2, and ACOT4 were identified as the highest scoring core genes related to fatty acid metabolism in DLBCL (**B**)

A total of 24 fatty acid metabolism related genes were screened in the DLBCL dataset GSE56315 and GSE25638, of which 22 were upregulated (Fig. 1A) and 2 were downregulated (Fig. 1B). 24 fatty acid metabolism related genes are mainly enriched in the GO pathway, including Monocarboxylic acid metabolic process, Unsaturated fatty acid metabolic process, Sulfur compound metabolic process, Lipid catabolic process, and Oxidoreductase activity (Fig. 1C).

### Hub gene analysis related to key fatty acid metabolism in DLBCL

Build a PPI network in the STRING database and visualize it using Cytoscape (Figs. 2A-B). Use the MCODE plugin to filter critical subnets. Identified as the highest scoring fatty acid related core gene in DLBCL. SLC27A2, MGLL, ACOT2, and ACOT4 were identified as the highest scoring core genes related to fatty acid metabolism in DLBCL. To verify the expression differences of the four hub genes, differential validation analysis was conducted in the TCGA-DLBCL and GTEx datasets. Compared with the control group, SLC27A2 showed significant differences (FC > 1.5 and P < 0.05) in the DLBCL group (Fig. S1).

### Cell cycle experiment

**Fig. 3:**
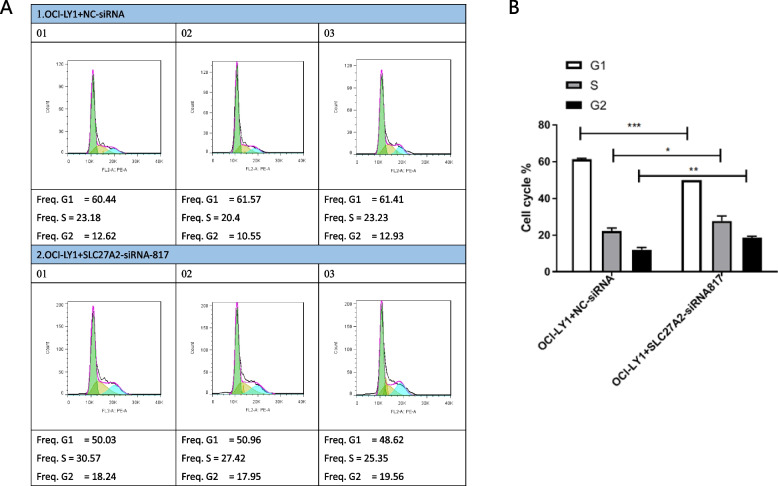
DLBCL Cell Cycle Experiment. The cell cycle results showed that compared with the OCI-LY1 + SLC27A2-siRNA-817 group, the G1 phase cells were significantly reduced, the S phase cells were significantly increased, and the G2 phase cells were significantly increased (**A**, **B**). Therefore, low expression of SLC27A2 significantly promoted the progression of the DLBCL cell cycle

The qPCR results showed that compared with the control group, the cells transfected with SLC27A2 siRNA3 had the most significant interference effect on SLC27A2 (Fig. S2). Therefore, the cells transfected with SLC27A2 siRNA3 were selected as the cell model for SLC27A2 interference and named SLC27A2 siRNA-817. The cell cycle results showed that compared with the OCI-LY1 + SLC27A2-siRNA-817 group, the G1 phase cells were significantly reduced, the S phase cells were significantly increased, and the G2 phase cells were significantly increased (Fig. 3A-B). Therefore, low expression of SLC27A2 significantly promoted the progression of the DLBCL cell cycle.

### Cell apoptosis experiment

**Fig. 4:**
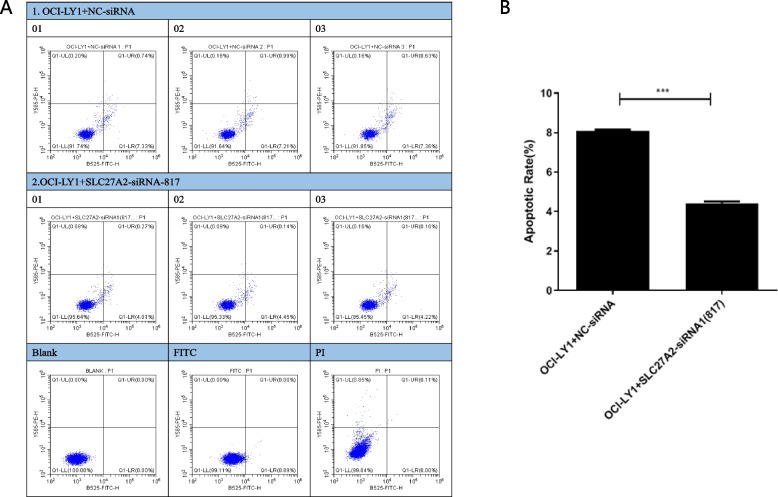
DLBCL cell apoptosis experiment. The results of cell apoptosis showed that compared with the OCI-LY1 + SLC27A2-siRNA-817 group, the cell apoptosis rate was significantly reduced (**A**, **B**). Therefore, low expression of SLC27A2 significantly inhibited the apoptosis of DLBCL cells

The results of cell apoptosis showed that the OCI-LY1 + SLC27A2-siRNA-817 group showed a significant decrease in the apoptosis rate compared to the OCI-LY1 + NC siRNA group cells (Fig. 4A-B). Therefore, low expression of SLC27A2 significantly inhibited the apoptosis of DLBCL cells.3.5. Validation of expression analysis of key Hub genes in AML.

To verify the expression differences of the four hub genes, differential validation analysis was conducted in the GSE30029, TCGA-LAML, TCGA-DLBCL and GTEx datasets. Compared with the control group, SLC27A2 showed significant differences (P < 0.05) in the DLBCL and AML group (Fig. S3).

### Correlation analysis of clinical information between SLC27A2 and DLBCL and AML

**Fig. 5:**
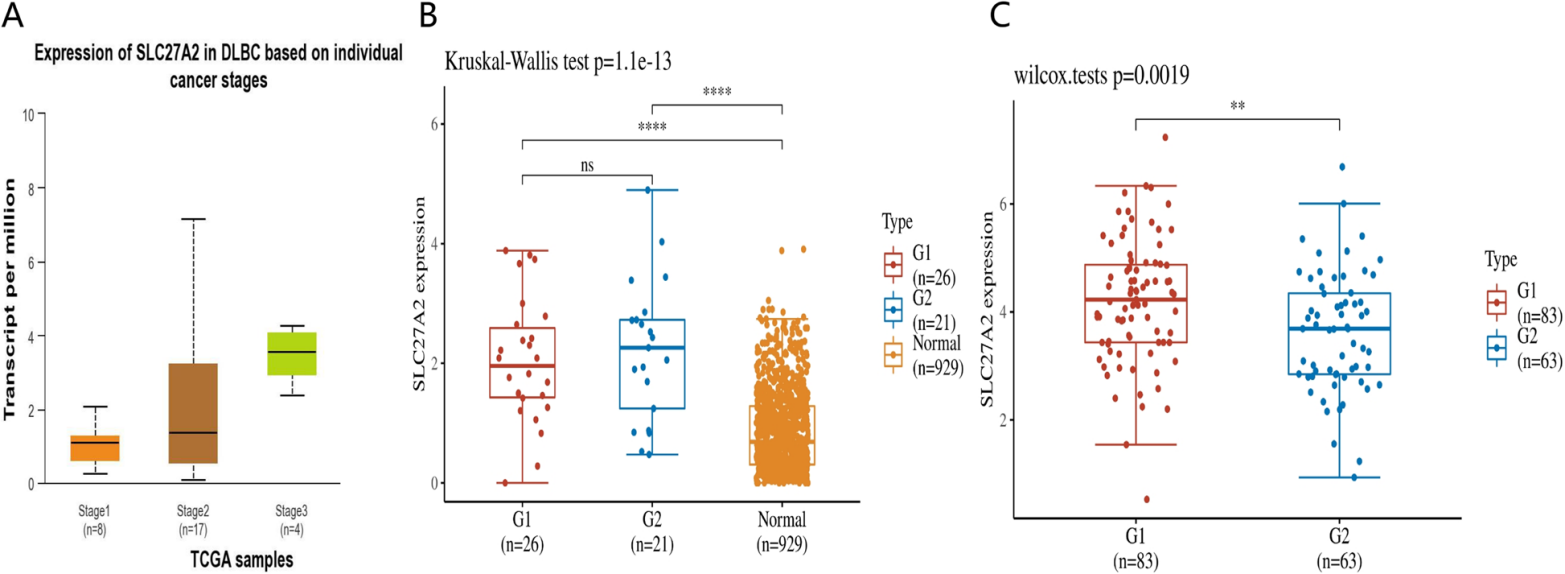
Correlation analysis of clinical information between SLC27A2 and DLBCL and AML**.** SLC27A2 showed a significant upward trend in stages 1, 2, and 3 (**A**), with the worst prognosis in stage 3 and the highest expression of SLC27A2. In DLBCL, SLC27A2 expression levels are highest in individuals aged over 60 years (G2) (**B**), while in AML, SLC27A2 expression levels are lowest in individuals aged over 60 years (G2) (**C**)

SLC27A2 showed a significant upward trend in stages 1, 2, and 3 (Fig. 5A), with the worst prognosis in stage 3 and the highest expression of SLC27A2. In DLBCL, SLC27A2 expression levels are highest in individuals aged over 60 years (G2) (Fig. 5B), while in AML, SLC27A2 expression levels are lowest in individuals aged over 60 years (G2) (Fig. 5C). Elderly DLBCL and elderly AML have a worse prognosis compared to younger DLBCL and younger AML, therefore SLC27A2 is a protective factor for hematological tumors.

### Correlation between SLC27A2 and immune cell infiltration

**Fig. 6:**
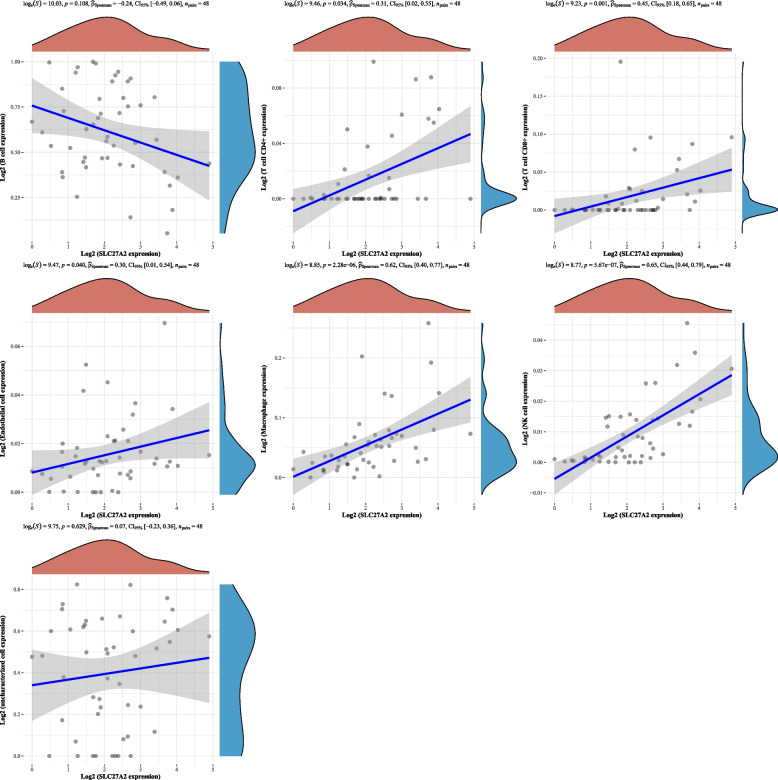
Correlation analysis between immune microenvironment and SLC27A2 in DLBCL**.** Immune microenvironment analysis showed that SLC27A2 was significantly positively correlated with T cell CD4 + , T cell CD8 + , endothelial cells, macrophages, and NK cells in DLBCL

**Fig. 7:**
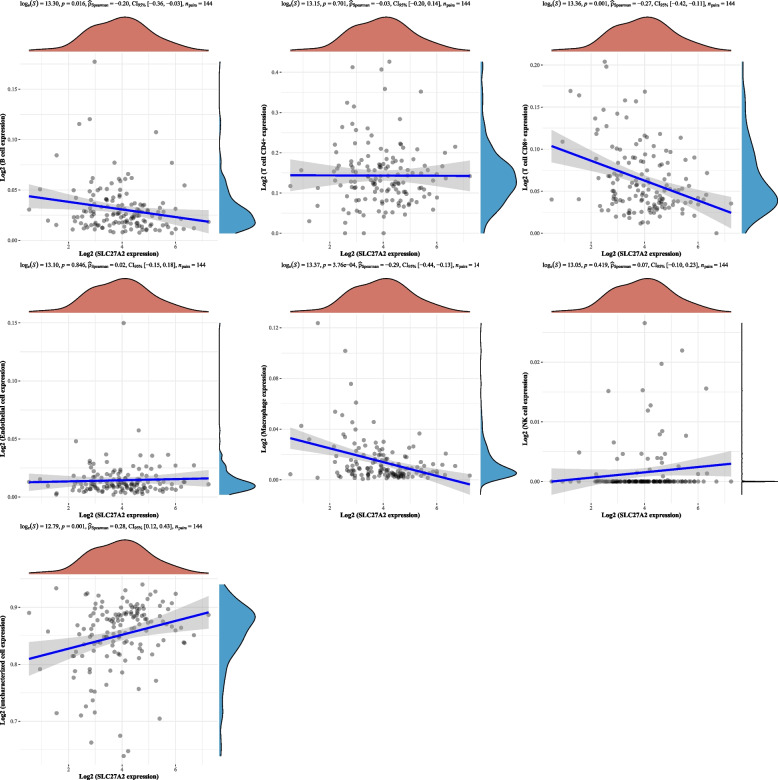
Correlation analysis between immune microenvironment and SLC27A2 in AML. In AML, there is a significant negative correlation between SLC27A2 and B cells, T cell CD8 + , and macrophages

Immune microenvironment analysis showed that SLC27A2 was significantly positively correlated with T cell CD4 + , T cell CD8 + , endothelial cells, macrophages, and NK cells in DLBCL (Fig. 6). In AML, there is a significant negative correlation between SLC27A2 and B cells, T cell CD8 + , and macrophages (Fig. 7). SLC27A2 participates in the immune process of hematological tumors through T cell CD8 + and macrophages, In DLBCL, patients with low expression of SLC27A2 have poorer prognosis, while in AML, patients with high expression of SLC27A2 have poorer prognosis (Fig. S4). SLC27A2 has opposite significance with immune cell infiltration in DLBCL and AML, indicating that SLC27A2 is a protective factor in hematological tumors [26].

### Functional pathway of SLC27A2 in DLBCL and AML

The GSEA results showed that the high expression group of SLC27A2 in DLBCL is mainly enriched in GO pathways, such as Fatty acid synthesis activity, Immune response to tumor cell, Mitotic DNA replication, Positive regulation of Immune response to tumor cell, and Regulation of chronic inflammatory response. Therefore, SLC27A2 is mainly involved in fatty acid related pathways in the pathological mechanism of DLBCL Tumor cell immune response and cell cycle related pathways (Fig. S5A). The GESA results indicate that low expression of SLC27A2 is mainly involved in the immune pathway (immune response-activating cell surface receptor signaling pathway and immune response-activating signal transduction) of AML (Fig. S5B).

### Protein interaction analysis of the SLC27A2 in DLBCL and AML

**Fig. 8:**
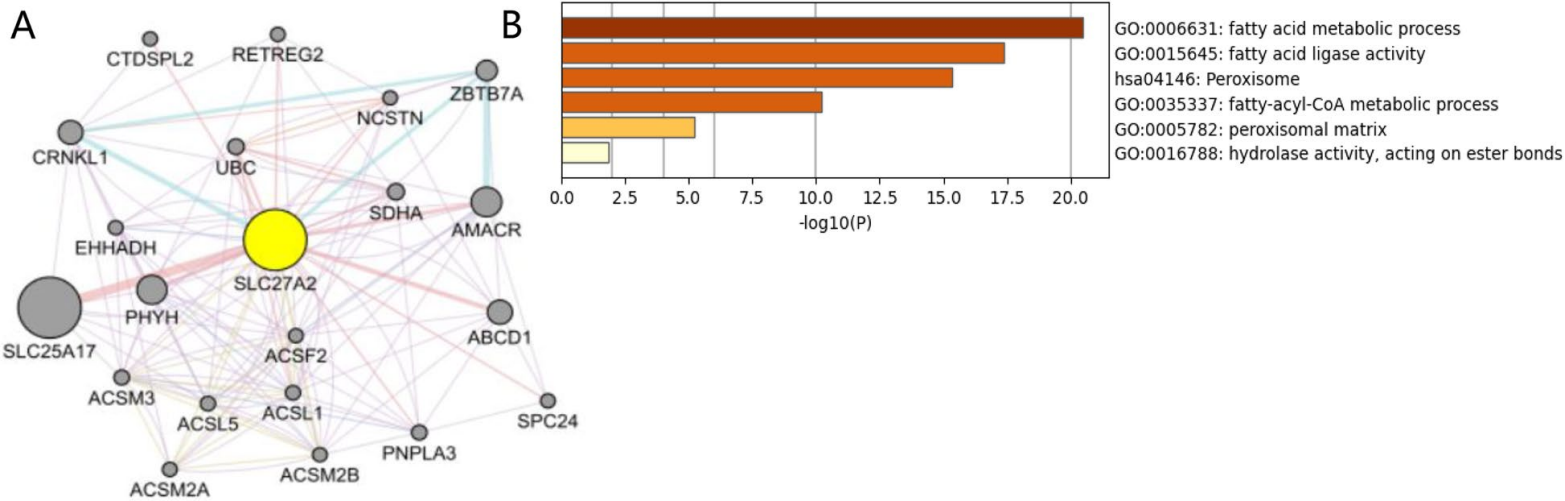
Protein Interaction Analysis of the Key Hub Gene SLC27A2 in DLBCL. A 21 gene PPI network centered on SLC27A2 was constructed using GeneMANIA (**A**). GO functional enrichment and KEGG pathway analysis were performed on these 21 genes. The significantly enriched GO terms include Fatty acid metabolic process, Fatty acid ligase activity, and Fatty acid CoA metabolic, while the significantly enriched KEGG pathway is Peroxisome (**B**)

A PPI network with 21 genes centered around SLC27A2 was constructed using GeneMANIA (Fig. 8A). GO functional enrichment and KEGG pathway analysis were performed on these 21 genes. The significantly enriched GO terms include Fatty acid metabolic process, Fatty acid ligase activity, and Fatty acid CoA metabolic, while the significantly enriched KEGG pathway is Peroxisome (Fig. 8B).

## Discussion

Fatty acid metabolism related genes play an important role in the progression of various cancers, and their role in DLBCL remains to be explored [13]. In this study, we systematically evaluated the role of fatty acid metabolism related genes in DLBCL and found that 22 differentially expressed fatty acid metabolism related genes were mainly enriched in the Monocarboxylic acid metabolic process, Unsaturated fatty acid metabolic process, Sulfur compound metabolic process, Lipid catalytic process, and Oxidoreductase activity pathways, which are mainly related to lipid metabolism Energy metabolism and oxidation are related [27]. Subsequently, we selected four key hub genes for further immune microenvironment research. The study found that changes in the expression level of SLC27A2 were significantly positively correlated with T cell CD4 + , Neutrophils, and Dendritic Cells, and negatively correlated with purity. In addition, patients with low expression of SLC27A2 had poor prognosis, and cell experiments confirmed that low expression of SLC27A2 promoted tumor cell cycle progression and inhibited cell apoptosis. Therefore, this study may provide valuable clues for the treatment of DLBCL patients.

In recent years, studies have found that SLC27A2 plays an important role in maintaining intracellular lipid balance, as well as in non-alcoholic fatty liver disease and renal fibrosis [28, 29]. In addition, it has been found that SLC27A2 is widely involved in the development of cancer. For example, blocking FATP2 in melanoma cells in the aging microenvironment can inhibit their lipid accumulation, and SLC27A2 specific inhibitors can slow down tumor growth, making SLC27A2 a beneficial target for targeted treatment of melanoma cells [30]. Research has shown that SLC27A2 induces cisplatin resistance in lung cancer stem cells through negative regulation of downstream signaling pathways, and the decrease in SLC27A2 is associated with chemotherapy response and poor patient survival [26]. In a recently published study, Feng et al. confirmed that upregulation of SLC27A2 expression in differentiated thyroid cancer can promote tumor proliferation and migration [31]. Meanwhile, SLC27A2 has also been found to be associated with the progression of ovarian cancer and renal clear cell carcinoma [32, 33]. Various studies have shown that SLC27A2 has outstanding therapeutic potential in various tumors, but there are no relevant reports on its mechanism of action in DLBCL. Our study found that SLC27A2 mainly plays a role in DLBCL through the Fatty acid metabolic process, Fatty acid ligase activity, Fatty acid CoA metabolic, and Peroxisome pathways. Therefore, SLC27A2 mainly plays a role in fatty acid metabolism, energy metabolism, and oxidation. Fatty acid metabolism and oxidation promote energy production [34], and SLC27A2 may affect the cycle and apoptosis of DLBCL cells by regulating energy metabolism. Fatty acid synthesis provides cell membranes and other key lipid cell structures for immune cell proliferation and is also necessary for the differentiation and function of inflammatory macrophages. In addition, the abnormal accumulation of fatty acids in tumor infiltrating myeloid cells has been demonstrated to tilt these immune cells towards immunosuppressive and anti-inflammatory phenotypes through metabolic reprogramming [35]. Therefore, SLC27A2 may directly participate in intracellular signaling and immune cell regulation mechanisms through fatty acid metabolism. Abnormal fatty acid metabolism is believed to be closely related to tumor cell cycle and apoptosis [36], and our study confirms this. Therefore, the abnormal expression of SLC27A2 leads to abnormal fatty acid metabolism in patients, thereby promoting the progression of tumor cell cycle and inhibiting cell apoptosis.

Inevitably, this study has several limitations. Part of the data in this study is from a public database and is retrospective. The clinical information data available for DLBCL patients is limited, so the clinical parameters analyzed in this study are not comprehensive. Secondly, the cell experiments involved in this study are limited to the DLBCL-GCB type, and different subtypes of DLBCL are highly heterogeneous in genetic characteristics and immune microenvironment. Therefore, further research is needed on the different subtypes of DLBCL. The upstream and downstream interaction mechanisms and immune effects of SLC27A2 in DLBCL have not been fully elucidated in the study, and further in-depth research is needed on the mechanism of action of SLC27A2 in DLBCL. Anyway, our results provide the first comprehensive report on the mechanism of action of fatty acid metabolism related genes in the occurrence, development, and immune microenvironment of DLBCL, and for the first time validate the potential mechanism of action of the key gene SLC27A2 in DLBCL through cell experiments.

## Conclusion

In summary, we conducted a comprehensive analysis of the fatty acid metabolism related gene SLC27A2 in hematological tumors for the first time. We identified SLC27A2 as a key gene involved in DLBCL and AML immunity through immune microenvironment analysis and GSEA analysis, and validated the effect of SLC27A2 on the DLBCL cycle through cell experiments. Clinical correlation analysis revealed SLC27A2 as a potential protective factor for DLBCL and AML. Therefore, we believe that our research findings can provide a theoretical basis for improving the treatment of DLBCL patients with AML.

### Supplementary Information

**Supplementary Material 1.**

## Data Availability

Gene expression microarray data sets, including GSE30029, GSE56315, GSE25638 and GSE181063, are downloaded from the Gene Expression Integrated Database (GEO) (https://www.ncbi.nlm.nih.gov/geo/).

## Abbreviations

AML/LAML: Acute myeloid leukemia
CHOR: Cyclophosphamide, Hydroxydaunomycin, Oncovin and Prednisone
DLBC/DLBCL: Diffuse large B cell lymphoma
FFPE: Formalin fixed paraffin embedded
GEO: Gene expression comprehensive database
GTEx: Genotype-tissue expression
GO: Gene Ontology
GSEA: Geneset enrichment analysis
KEGG: Kyoto Encyclopedia of Genes and Genomes
TME: Tumor microenvironment
TCGA: The cancer genome atlas
