# Supplementary material for: SLC27A2 is a potential immune biomarker for hematological tumors and significantly regulates the cell cycle progression of diffuse large B-cell lymphoma
Source: BMC Med Genomics. 2024 Apr 25;17:105. doi: 10.1186/s12920-024-01853-3 (PMC11046844; doi:10.1186/s12920-024-01853-3)
Supplement: Supplementary file 1 — Supplementary Material 1. [file 12920_2024_1853_MOESM1_ESM.docx]

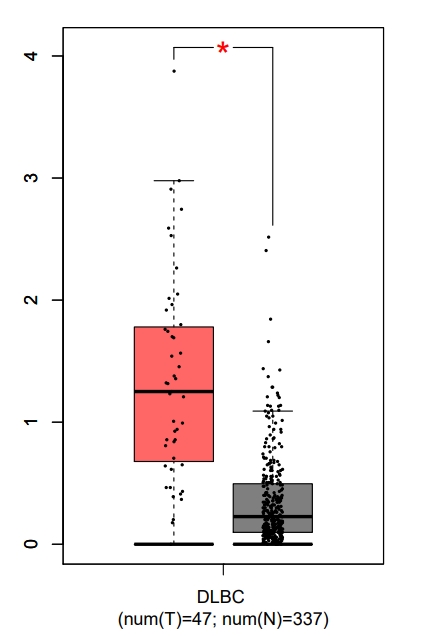


**Figure S1 The TCGA-DLBC to validate the expression of SLC27A2.**

Compared with the control group sample, SLC27A2 were significantly expressed in DLBCL. Red represents the disease group, black represents the health group (*P<0.05 and FC>1.5).


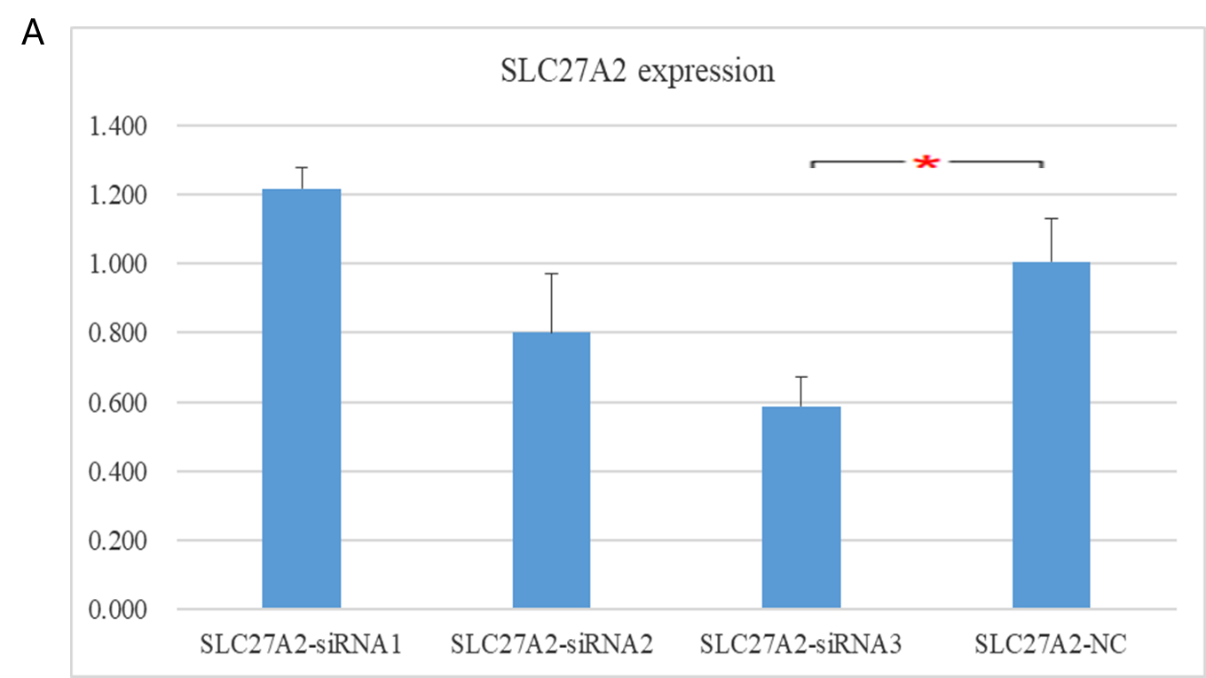


**Figure S2 qPCR validation of the interference effect of siRNA transfection on SLC27A2 expression in DLBCL cells**

(A) The qPCR results showed that compared with the control group, the SLC27A2 siRNA3 transfected cells had the most significant interference effect on SLC27A2


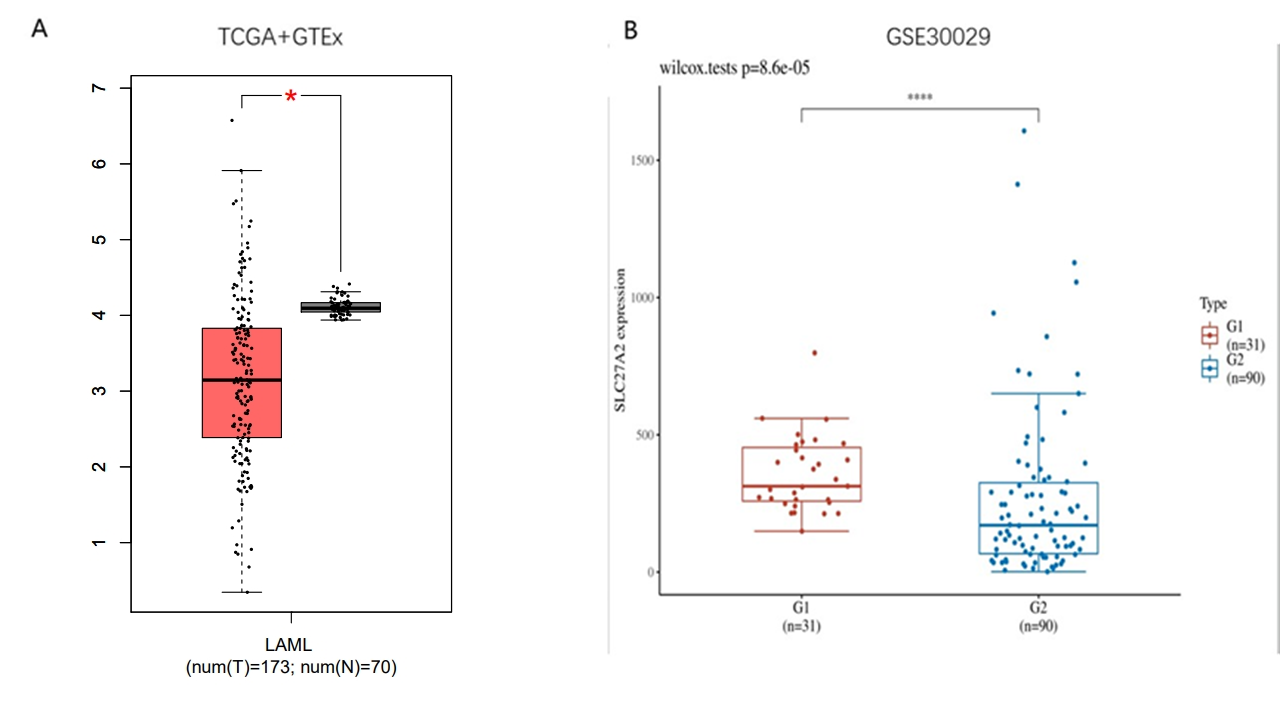


**Figure S3 The TCGA-LAML and GSE30029 to validate the expression of SLC27A2.**

(A) Compared with the control group sample, SLC27A2 were significantly expressed in AML. Red represents the disease group, black represents the health group (*P<0.05 and FC>1.5). (B) Compared with the control group sample, SLC27A2 were significantly expressed in AML. G1 represents the health group, G2 represents the disease group (*P<0.05 and FC>1.5).


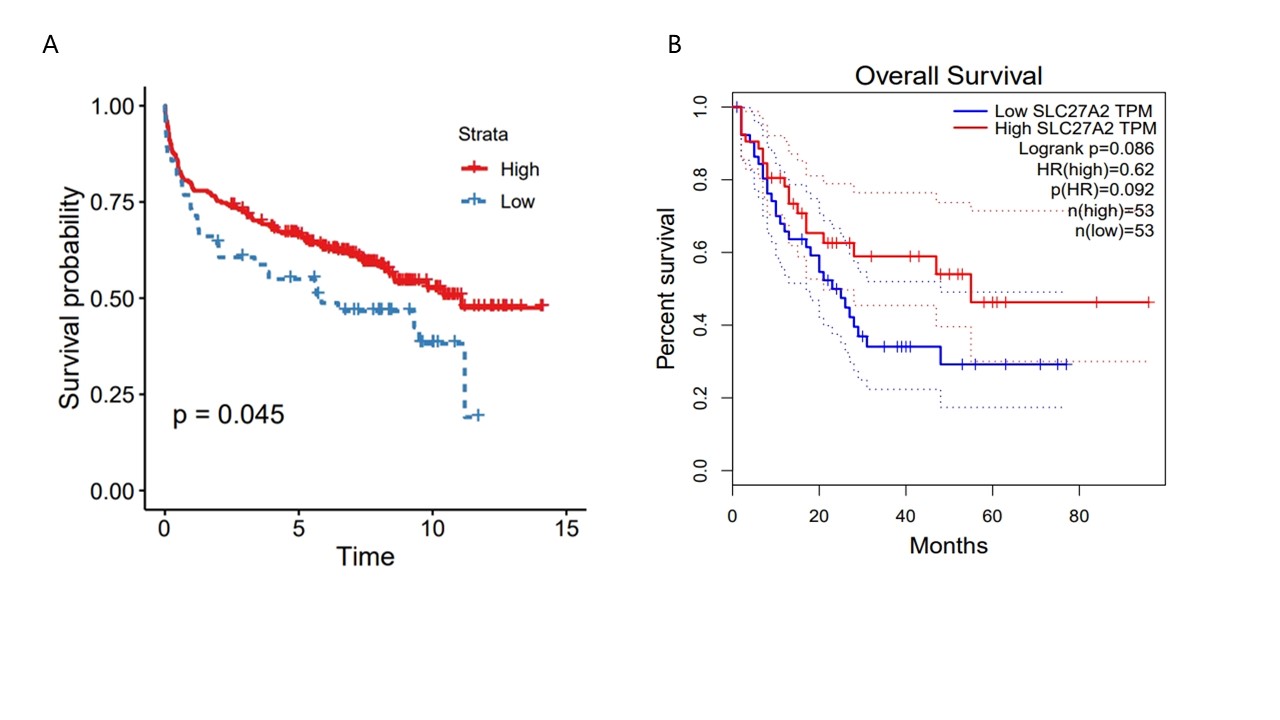


**Figure S4 The R 4.2.1 tool was used to analyze the raw data of microarrays to evaluate the correlation between gene expression and prognosis.**

(A) The Kaplan Meier survival curve shows that compared to patients with high expression of SLC27A2, patients with low expression have poor prognosis in DLBCL.

(B) The Kaplan Meier survival curve shows that compared to patients with low expression of SLC27A2, patients with high expression have poor prognosis in AML.


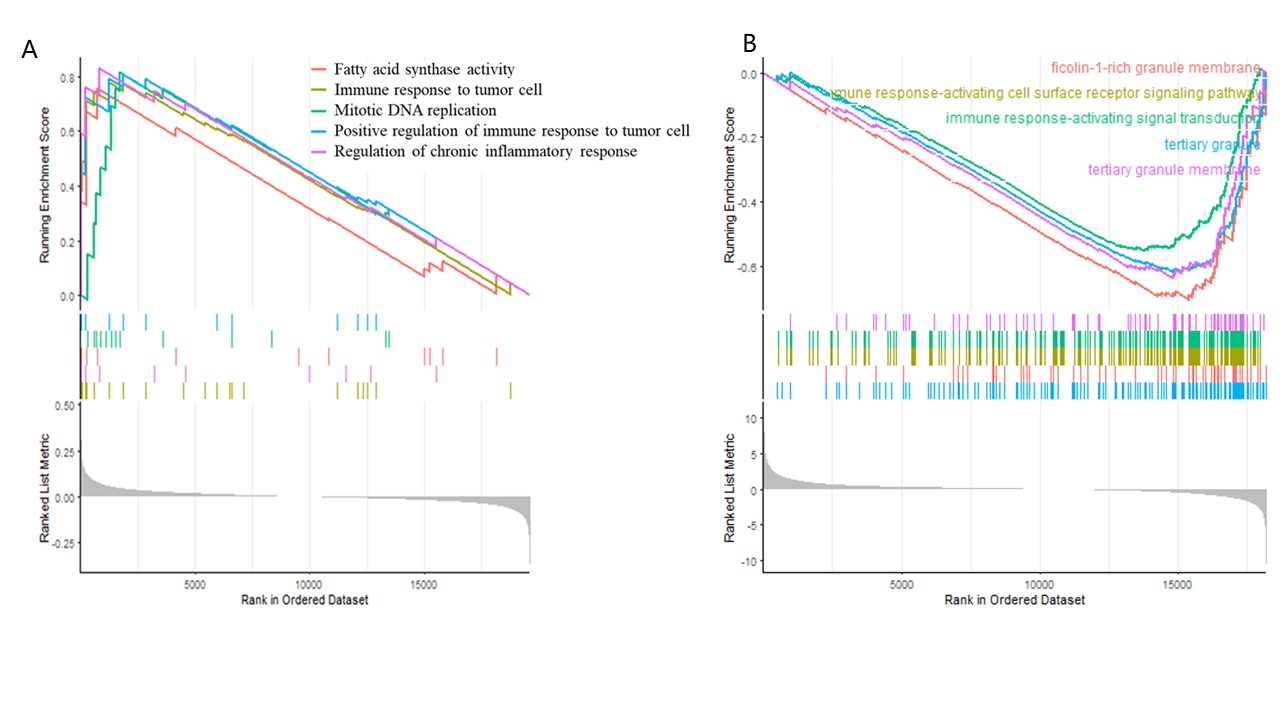


**Figure S5 Enrichment Analysis (GSEA) Study on the Biological Signal Pathway of High and Low Expression of SLC27A2 in DLBCL and AML.**

(A) The GSEA results showed that the high expression group of SLC27A2 in DLBCL mainly enriched GO pathways, such as Fatty acid synapse activity, Immune response to tumor cell, Mitotic DNA replication, Positive regulation of Immune response to tumor cell, and Regulation of chronic inflammatory response.

(B) The GESA results indicate that low expression of SLC27A2 is mainly involved in the immune pathway of AML.
